# Synthesis and Broadband Spectra Photocatalytic Properties of Bi_2_O_2_(CO_3_)_1−*x*_S*_x_*

**DOI:** 10.3390/ma11050791

**Published:** 2018-05-14

**Authors:** Junping Ding, Huanchun Wang, Haomin Xu, Lina Qiao, Yidong Luo, Yuanhua Lin, Cewen Nan

**Affiliations:** 1 State Key Laboratory of New Ceramics and Fine Processing, School of Materials Science and Engineering, Tsinghua University, Beijing 100084, China; djp15@mails.tsinghua.edu.cn (J.D.); huanchwang@163.com (H.W.); xuhm13@mails.tsinghua.edu.cn (H.X.); qln13@mails.tsinghua.edu.cn (L.Q.); ydluozd@163.com (Y.L.); cwnan@mail.tsinghua.edu.cn (C.N.); 2 China Astronaut Research and Training Center, Beijing 100094, China; 3 High-Tech Institute of Xi’an, Xi’an 710025, China

**Keywords:** Bi_2_O_2_CO_3_, Bi_2_O_2_(CO_3_)_1−*x*_S*_x_*, broadband spectra, photocatalysis

## Abstract

High efficiency photocatalyst Bi_2_O_2_(CO_3_)_1−*x*_S*_x_* was synthesized conveniently with chemical bath precipitation using Bi_2_O_2_CO_3_ as the precursor. The microstructures of the samples are systematically characterized by X-ray diffraction (XRD), scanning electron microscopy (SEM), high resolution transmission electron microscopy (HRTEM), X-ray photoelectron spectroscopy (XPS), ultraviolet photoelectron spectroscopy (UPS) and UV-Vis spectroscopy; the optical and photocatalytic properties are carefully tested as well. The content of *S*, which was tuned through the controlling of the precipitation process, was verified to have an intense effect over the photocatalytic properties. A nearly saturated *S* ratio and the best photocatalytic performance were observed in specimens with the most *S* content. Our study reveals that, with negligible influence of the morphology and crystal structure, Bi_2_O_2_(CO_3_)_1−*x*_S*_x_* possessed a broadened optical absorption regionfromultraviolet to visible light, and enhanced photocatalytic activity in comparison to precursor Bi_2_O_2_CO_3_ in photocatalytic degradation of Congo Red aqueous solution.

## 1. Introduction

Semiconductor photocatalysis has attracted increasing attention because of the capability of harvesting the solar energy to eliminate environmental pollutants [1,2,3,4,5,6,7]. Among various semiconductors, some Aurivillius type bismuth-based oxide semiconductor materials such as BiOX (X = Cl, Br, I), BiVO_4_ and Bi_2_WO_6_ have been widely used in photocatalysis [8,9,10,11,12,13,14]. 

Bismuth-based layered-structure compounds have a unique crystal structure and band structure. Hybridisation between 6s electrons of Bi and 2p electrons of O form chemical bonds which are stronger than those between Bi and other nonmetallic atoms (such as chalcogen), leading to a particularly stable (Bi-O)^+^ layer. A series of Bi-based layered-structural photocatalytic materials of various band gap widths from 3.2 eV (e.g., BiOCl [15]) to 1.12~1.5 eV (e.g., Bi_2_O_2_S [16,17]) can be obtained by combining the (Bi-O)^+^ layer with different anion layers. In addition, p-type (BiCuSO or the like) or n-type (Bi_2_O_2_CO_3_, etc.) semiconductor materials can be obtained by adjusting the anion layer. Therefore, the different Bi-based oxide composite structure can not only control and broaden the range of light absorption of the catalyst, but also may form a hetero structure such as p-n junction.

Recently, Bi_2_O_2_CO_3_, which is a member of the Aurivillius-type family and composed of [Bi_2_O_2_]^2+^ layers interleaved by CO_3_^2−^ layers [18,19], has attracted growing concern because of its photocatalytic ability to decompose organic pollutants in liquid phase and NO in gaseous phase [20,21,22]. Its unique layered structure, resulting in a large internal electrostatic field and asymmetric polarization effect, contributes to the separation of photogenerated electron-hole pairs [23,24]. However, the application of Bi_2_O_2_CO_3_ in photodegradation is strongly limited by its large band gap (~3.3 eV). To overcome this limitation, many methods have been developed, such as the fabrication of heterojunctions such as BiVO_4_/Bi_2_O_2_CO_3_, Bi_2_S_3_/Bi_2_O_3_/Bi_2_O_2_CO_3_ [25,26,27], noble metal deposition [28], elemental doping [29], and morphological modulation [30].

In this paper, we have synthesized S-doped Bi_2_O_2_(CO_3_)_1−*x*_S*_x_* by chemical bath precipitation, using Bi_2_O_2_CO_3_ as the precursor, through the controlling of the precipitation process to have an intense effect over the photocatalytic properties. A nearly saturated *S* ratio and the best photocatalytic performance were observed in specimens with the most *S* content. With a negligible influence of the morphology and crystal structure, the optical absorption of Bi_2_O_3_CO_3_ was extended from the ultraviolet (UV) to the visible region. The photocatalytic degradation of Congo Red showed that Bi_2_O_2_(CO_3_)_1−*x*_S*_x_* exhibited enhanced photoactivity in comparison to the precursor powder.

## 2. Results and Discussion

### 2.1. Synthetic Bi_2_O_2_(CO_3_)_1−x_S_x_

**Figure 1:**
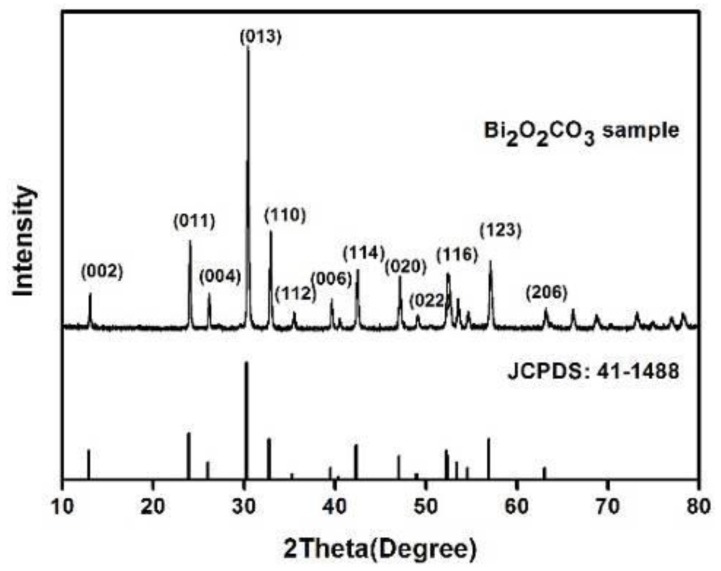
XRD pattern of hydrothermal synthesised Bi_2_O_2_CO_3_.

Figure 1 shows the XRD(X-ray diffraction) pattern of the Bi_2_O_2_CO_3_ powder prepared by hydrothermal method, together with a reference pattern of tetragonal Bi_2_O_2_CO_3_ (JCPDS: 41−1488). No second phase can be found, and the sharp peaks indicate well-developed crystallinity. The preparation process of Bi_2_O_2_CO_3_ can be summarized in Equations (1)–(3). CO_3_^2−^ forms through a hydrolysis reaction between (NH_2_)_2_CO and H_2_O. Bi_2_O_3_ is also strongly hydrolyzed with water to produce (Bi_2_O_2_)^2+^. The produced (Bi_2_O_2_)^2+^ and CO_3_^2−^ then react to generate Bi_2_O_2_CO_3_. 

(NH_2_)_2_CO + 2H_2_O → 2NH_3_^+^ + CO_3_^2−^ (1)

Bi_2_O_3_ + 2H_2_O →(Bi_2_O_2_)^2+^ + 2OH^+^ (2)

(Bi_2_O_2_)^2+^ + CO_3_^2−^→Bi_2_O_2_CO_3_ (3)

**Figure 2:**
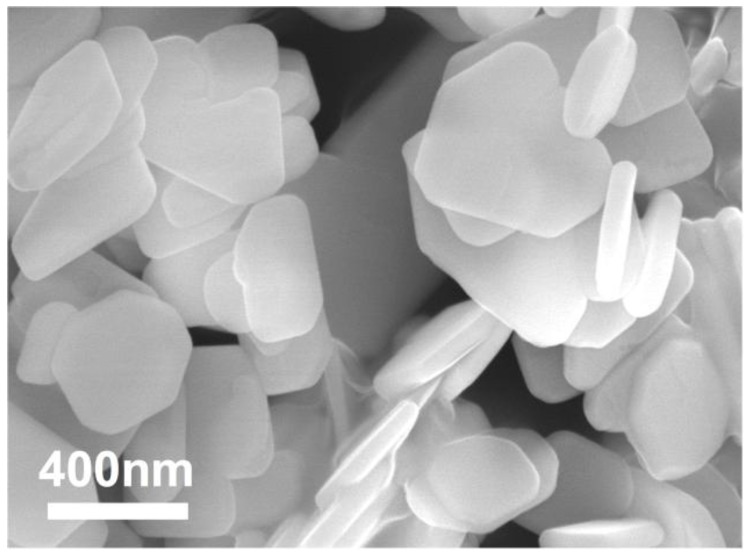
SEM photograph of hydrothermal synthesised Bi_2_O_2_CO_3_.

**Figure 3:**
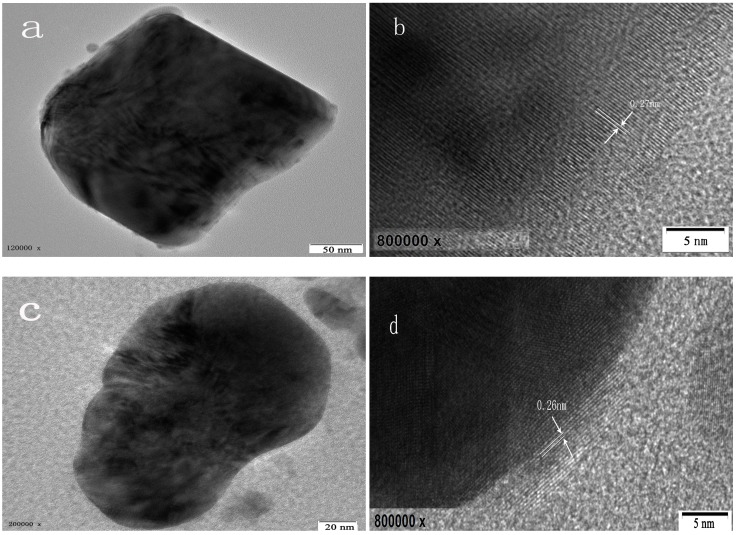
HRTEM images of M0 (**a**); (**b**) and M20 (**c**); (**d**).

In addition, the percentage of crystallinity and the BET (Brunauer–Emmett–Teller) specific surface area of the samples with a S:Bi_2_O_2_CO_3_ ratio n equals to 0, 0.01, 0.02, 0.05, 0.10 and 0.20 (marked as M0, M1, M2, M5, M10 and M20, respectively) are shown in Table 1. There are no significant changes in their percentage of crystallinity, while samples of M5 and M10 displaylarger specific surface areas than that of other samples, which could lead to theexposure of more active sites for the photocatalytic experiment. The scanning electron microscopy (SEM) photograph and the high resolution transmission electron microscopy (HRTEM) images of the powder are shown in Figure 2 and Figure 3. The morphology of the particles are nano-sized flakes of about 60–80 nm in thickness. In addition, the crystallinity of different samples calculated from the XRD results shows that *S* doping introduced defects in the Bi_2_O_2_CO_3_ and thus caused crystallinity change.

**Figure 4:**
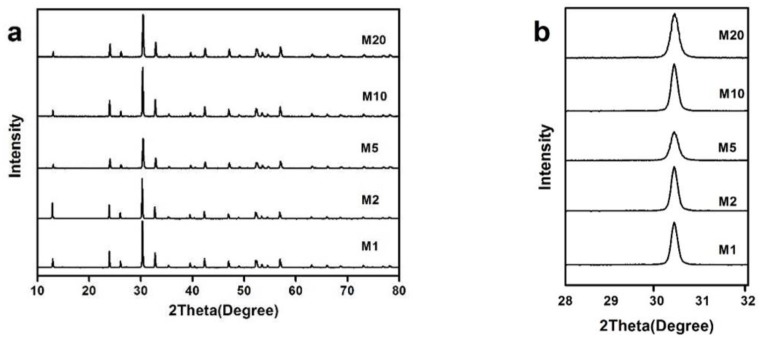
(**a**) XRD patterns of Bi_2_O_2_(CO_3_)_1−*x*_S*_x_* prepared by chemical bath precipitation; (**b**) the position of the (013) diffraction.

The XRD patterns of the samples prepared by the Na_2_S chemical bath precipitationare shown in Figure 4a. All diffraction peaks are consistent with Bi_2_O_2_CO_3_, indicating that chemical bath precipitation did not introduce a significant second phase. The intensity of the diffraction peak does not obviously decrease, and the products still have good crystallinity. The position of the (013) diffraction peak for different samples are shown in Figure 4b. No obvious influence of Na_2_S chemical precipitation on the crystal structure of Bi_2_O_2_CO_3_ can be found because the position of the peak (013) did not show an apparent shift according to XRD results.

**Figure 5:**
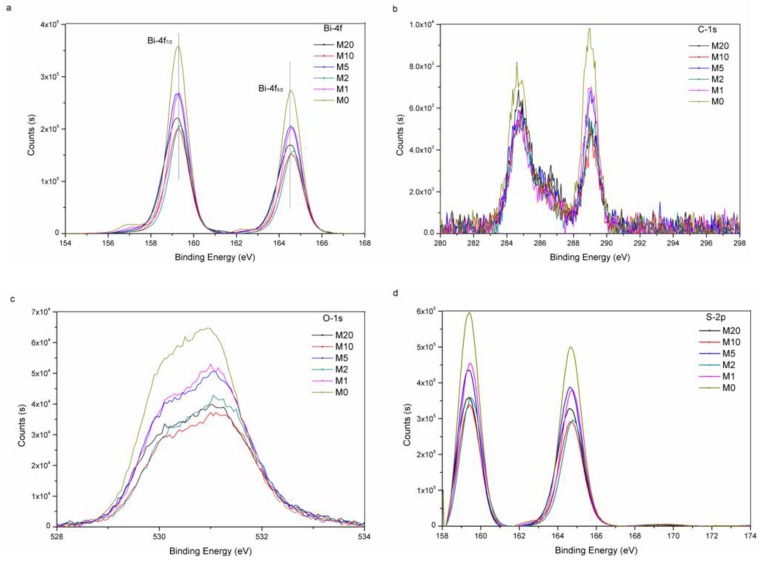
XPS spectra of M0, M1, M2, M5, M10 and M20, Bi-4f (**a**); C−1s (**b**); O−1s (**c**); and S-2p (**d**).

X-ray photoelectron spectroscopy (XPS) was utilized toobtain insights into the valence states and surface chemical compositions details of Bi_2_O_2_(CO_3_)_1−*x*_S*_x_*. As shown in Figure 5a, the XPS spectrum of Bi-4f shows two peaks at 159.05 and 164.35 eV, which belong to Bi-4f_7/2_ and Bi-4f_5/2_ energy levels, respectively. These two peaks are characteristic features of trivalent Bi in Bi_2_O_2_(CO_3_)_1−*x*_S*_x_* [31]. The two peaks at 284.7 eV and 288.8 eV in Figure 5b show that the existence form of C is CO_3_^2^^−^ [32]. In Figure 5c, the two peaks are at 530.5 eV and 531 eV, which belong to O energy levels in B-O and CO_3_^2−^, respectively [33]. In Figure 5d, the peak of S-2p is at the range of 158–166 eV, which shows that the existence form of *S* is S^2−^ [17]. On the other hand, the Bi-4f peak of M20 apparently shifts compared to M0 (Figure 5a), which proves that *S* takes place of CO_3_^2^^−^ partially [34].

**Figure 6:**
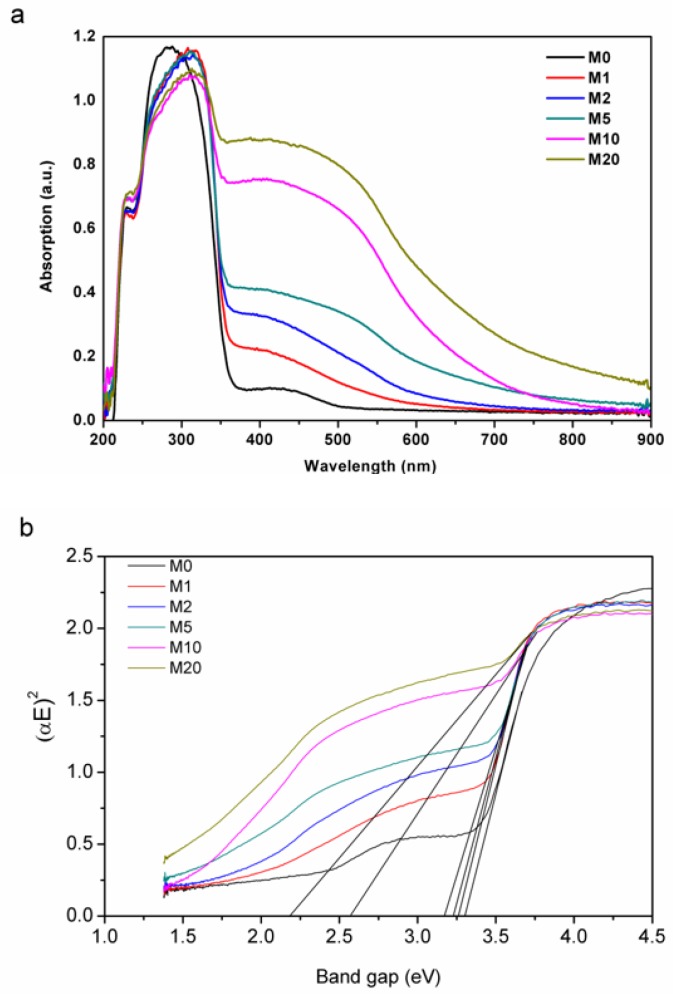
(**a**) UV-Vis diffuse reflectance spectra of the synthesized Bi_2_O_2_(CO_3_)_1−*x*_S*_x_*; (**b**) band gap fitting with K-M relation.

**Figure 7:**
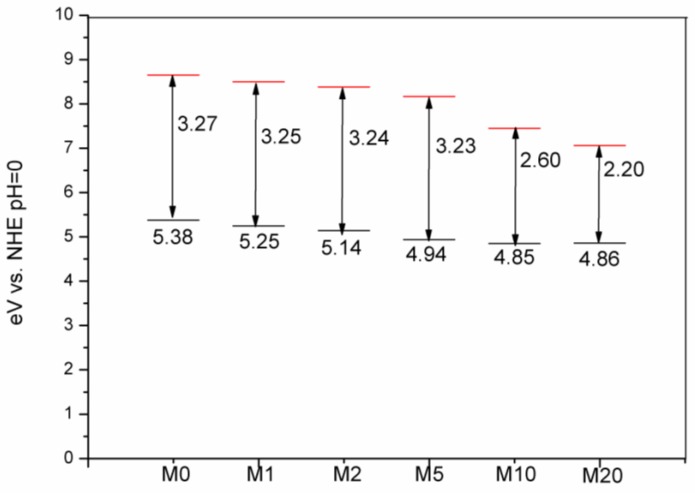
Energy levels of the conduction band minimum (E_CB_, red) and the valence band maximum (E_VB_, black) calculated at theoretical pH = 0 (V is voltage; NHE is normalhydrogen electrode potential).

Although Na_2_S chemical precipitation had no obvious influence on the crystal structure of Bi_2_O_2_CO_3_, the powder color was changed from white to yellow, and the color became darker as S: Bi_2_O_2_CO_3_ molar ratio *n* increased. The UV-Vis diffuse reflectance spectra are shown in Figure 6. Bi_2_O_2_CO_3_ has a strong absorption of UV light with wavelengths less than 360 nm and weak absorption to 400 nm~500 nm-wavelength-visible light due to defects and oxygen vacancy, which also explained the fact that Bi_2_O_2_CO_3_ could display visible light photocatalytic activity with the bandgap of 3.2 eV. With the introduction of *S*, the light absorption behaviour was significantly changed from M1 to M20. In particular, the absorption of visible light increased by about one order of magnitude. The band gap of Bi_2_O_2_CO_3_ without sulfur is fitted as 3.27 eV, and the introduction of *S* leads to the emergenceof a narrow band gapby lowering the conduction band position and meanwhile generating impurity levels [35,36]. The adsorption edge is around 380 nm. With the increase of *S* content, defects and oxygen vacancies increase, possibly due to point defects, and the fitted narrow band gap decreases from 3.25 to 2.20 eV. Energy levels of the valence band maximum (E_VB_) were measured by the ultraviolet photoelectron spectrometer at UV intensity 500 nW and energy levels of the conduction band minimum (E_CB_) were calculated by the bandgap. As shown in Figure 7, valence band edge position and conduction band edge position become more negative after the incorporation of sulfur into Bi_2_O_2_CO_3_.

**Figure 8:**
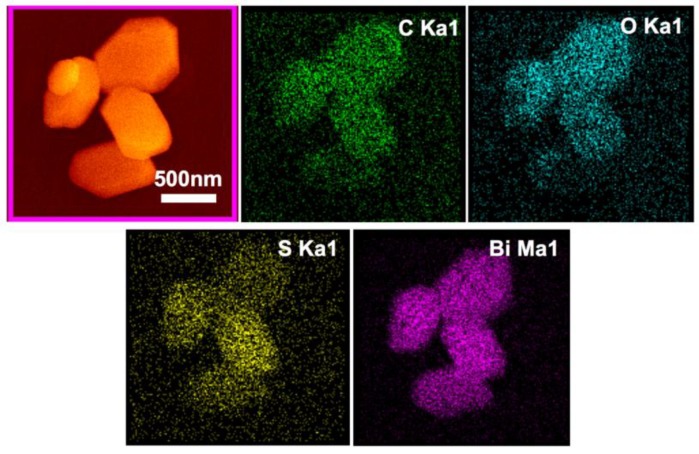
EDS element mapping details of M20 particles.

The SEM observation showed that chemical bath treatment had little influence on the morphology of the Bi_2_O_2_CO_3_ particles. EDS (energy dispersive spectroscopy) elemental mapping in Figure 8 revealed the homogeneous distribution of *S* on the particle surface, and no obvious segregation and aggregation can be seen among the particles. The quantitative elemental analysis results are shown in Table 2. The *S* content in samples M1~M20 increases with the increase of *S*: Bi_2_O_2_CO_3_ molar ratio *n*, but the *S* atom percentage (referring to Bi-content) is obviously smaller than *n* and becomes stable as *n* is greater than 0.10. This is consistent with the calculations about the surface adsorption of Bi_2_O_2_CO_3_ by Chang [34], who suggested that S^2−^ can be adsorbed in the oxygen vacancy of the (001) plane via the chemical bonding and reduce the surface energy. The calculation of the density of states near the Fermi level shows that the doping of *S* can introduce a new energy level in the energy band and reduce the band gap. The electron state density near the Fermi surface is more diffusive, which favours the migration of electrons and therefore improves the photocatalytic performance.

### 2.2. Ultraviolet-Visible Light Photocatalytic Properties of Bi_2_O_2_(CO_3_)_1−x_S_x_

**Figure 9:**
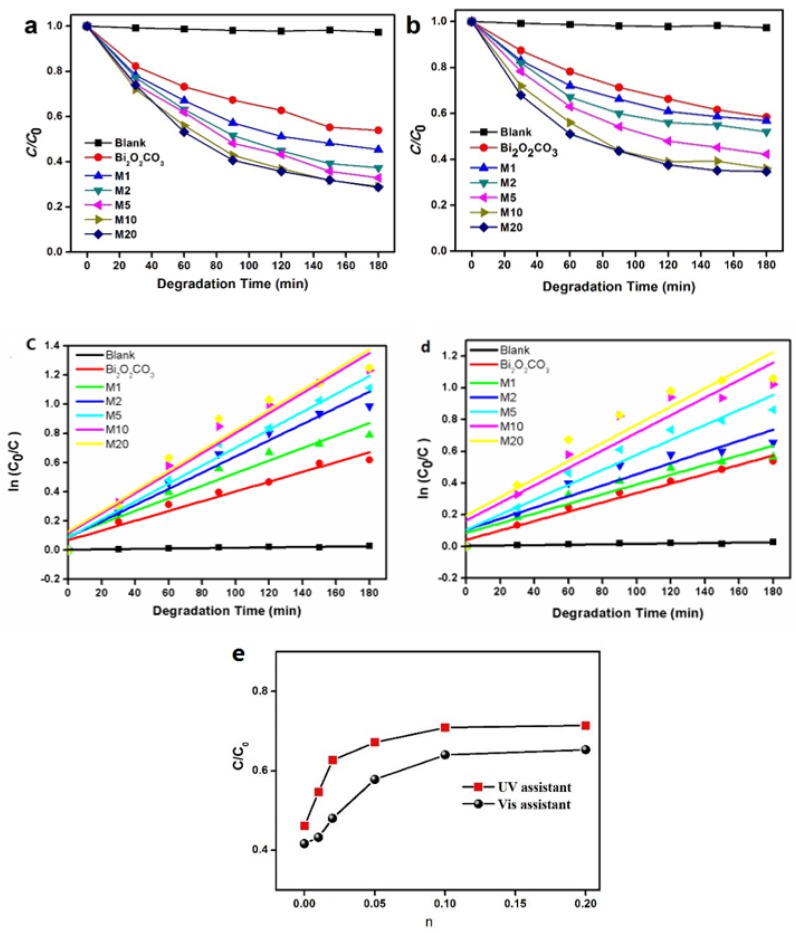
Degradation of Congo Red under the irradiation of (**a**,**c**) UV, and (**b**,**d**) visible light, with Bi_2_O_2_(CO_3_)_1−*x*_S*_x_*; (**e**) the degradation rate as a function of *n* (=S:Bi_2_O_2_CO_3_).

The photocatalytic activity of Bi_2_O_2_(CO_3_)_1−*x*_S*_x_* was characterised by photocatalytic degradation of Congo Red. As is shown in Figure 9, the introduction of *S* could improve the photocatalytic activity of Bi_2_O_2_CO_3_ under visible light and UV light. We measured the dye adsorption before switching on the light and normalized the concentrations, which made initial values of c/c_0_ equal to 1 for all samples. The operation temperature used was around 0 °C. With pure Bi_2_O_2_CO_3_, the Congo Red degrades by 41.6% under the irradiation of visible light for 3h, and by 46.1% under that of UV light, respectively. With the increase of molar ratio of S: Bi_2_O_2_CO_3_ from 0.01 to 0.1, the degradation rate increases to 64.2% and 70.1%, respectively. The further increase of *n*, however, cannot further remarkably increase the degradation rate. At the highest molar ratio of *S*(0.2), the photocatalytic activity of Congo Red was 65.3% and 71.4%, respectively, which was 1.57 and 1.55 times higher than that of Bi_2_O_2_CO_3_, respectively. The photo-degradation behavior of CR by use of Bi_2_O_2_(CO_3_)_1−*x*_S*_x_* obeys pseudo-first-order kinetics. This can be fitted by the Langmuir–Hinshewood model of ln(C_0_/C) = kt + A, where k is the reaction rate constant, t is the degradation time and the intercept A is the initial value of ln(C_0_/C), which means the dark adsorption of substrates. The *k* value of M1, M2, M5, M10 and M20 under UV light is 4.3 × 10^−3^ min^−^^1^, 5.5 × 10^−^^3^ min^−^^1^, 6.1 × 10^−^^3^ min^−^^1^, 6.8 × 10^−^^3^ min^−^^1^ and 6.9 × 10^−^^3^ min^−^^1^, respectively. The *k* value under visible light is 3.0 × 10^−^^3^ min^−^^1^, 3.5 × 10^−^^3^ min^−^^1^, 4.7 × 10^−^^3^ min^−^^1^, 5.5 × 10^−^^3^ min^−^^1^ and 5.9 × 10^−^^3^ min^−^^1^, respectively. The strong visible light sensitivity indicates higher utilization efficiency of solar light, making Bi_2_O_2_(CO_3_)_1−*x*_S*_x_* a superior photocatalyst than the commercial P25 TiO_2_, which has been reported to be hardly able to respond to visible light [37,38].

**Figure 10:**
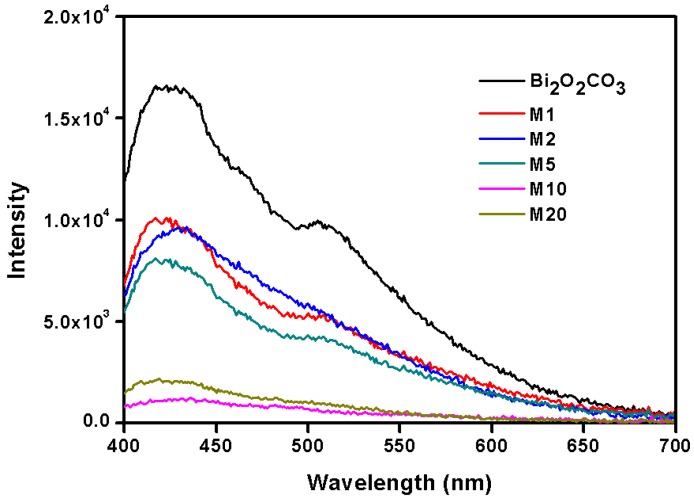
Photoluminescence spectra of Bi_2_O_2_(CO_3_)_1−*x*_S*_x_*.

Chang’s theoretical calculations [34] suggest that *S* can be easily captured and adsorbed by oxygen vacancies on the surface of Bi_2_O_2_CO_3_ as formed S^2−^ can partially substitute CO_3_^2−^ without forming a second phase, introducing a bend built-in electric field. At the same time, their experiments also confirmed that Bi_2_O_2_(CO_3_)_1−*x*_S*_x_* had higher conductivity and better carrier transport performance. The photoluminescence (PL) spectra of different S-substituted Bi_2_O_2_(CO_3_)_1−*x*_S*_x_* (Figure 10) show that samples M10 and M20 displayed weaker electron holes and recombination, indicating that the introduction of *S* can effectively suppress the carrier recombination. The stronger light absorption contributed by the smaller band gap means more photo-induced electron hole generation, and those electron holes showed better separation according to PL spectra. It is worthnoting that M20 has astronger light absorption and smaller bandgap than thatof M10, but that they presentnearly the same photocatalytic activity, which may be caused by the smaller specific surface area (Table 1) and slightlyweaker separation (Figure 10) of M20. Thus, all the three factors helped enhance the photocatalytic performance of Bi_2_O_2_CO_3_ in our samples under UV and visible light irradiation.

## 3. Materials and Methods

### 3.1. Preparation of the Precursor Powder Bi_2_O_2_CO_3_ via Hydrothermal Method

Three grams of urea (≥99.0%, Beijing Modern Orient Fine Chemistry Co. Ltd., Beijing, China) was dissolved in 60 mL of deionized water in a Teflon hydrothermal tank. 4.65 g Bi_2_O_3_ powder (99.99%, Aladdin Industrial Corporation, Shanghai, China) was then introduced into the solution. The hydrothermal tank was then tightly closed and kept in an oven at 180 °C for 12 h. After cooling down to room temperature, the precipitate was separated and washed with deionized water and ethanol several times and then dried in the oven at 70 °C.

### 3.2. Preparation of Bi_2_O_2_(CO_3_)_1−x_S_x_ by Chemical Bath Precipitation

Five suspensions of Bi_2_O_2_CO_3_ were prepared, each by dispersing 2.04 g of Bi_2_O_2_CO_3_ powder in 50 mL of deionized water with the help of ultrasonic stirring for 10 min. A certain amount (S:Bi_2_O_2_CO_3_ ratio *n*, equals to 0.01, 0.02, 0.05, 0.10 and 0.20, respectively) of 0.5 mol/L Na_2_S (≥98.0%, Shanghai Tongya Chemical Technology Co. Ltd., Shanghai, China) solution was introduced into the respective suspensions. After 8 h of further magnetic stirring at room temperature, the precipitates were separated and washed several times with deionized water and ethanol and dried at 70 °C. As-treated powders were numbered as M1, M2, M5, M10 and M20, respectively.

### 3.3. Characterization

Powder X-ray diffraction (XRD) was completed on a diffractometer (D8-Advance, Bruker, Billerica, MA, USA)using monochromatized Cu Kα (λ = 0.15418 nm) radiation with scanning speed of 3°/min. The morphologyof the sampleswerecarried out on a scanning electron microscope (JSM-7001F, JEOL, Tokyo, Japan) operating at a 5 kV and a field emissionelectron microscope (JEM-2100F, JEOL). The surface areas of specimens were tested on a automated gas sorption anslyser (Quantachrome, autosorb iQ2). The X-ray photoelectron spectroscopic (XPS) measurements were performed on a Thermo Fisher ESCALAB 250Xi instrument. A UV-Vis-NIR spectrometer (Lambda 950, PerkinElmer, Waltham, MA, USA) was used to measure UV-Vis diffuse reflectance spectra (DRS). Energy levels of the valence band maximum (E_VB_) were measured by the ultraviolet photoelectron spectrometer (AC-2, RIKEN KEIKI, Tokyo, Japan).

### 3.4. Photocatalytic Test

The photocatalytic activity of the prepared Bi_2_O_2_(CO_3_)_1−*x*_S*_x_* powder samples was evaluated by photodegrading Congo Red (CR, 100 mg/L) aqueous solution.The reason we chose this concentration is because it is proper to evaluate the change of the color. 0.16 g photocatalyst powder specimen was dispersed into 80 mL CR solution and stirred in the dark for 2 h to reach theadsorption–desorption equilibrium between the photocatalysts and organic dye molecules. Magnetic stirringanda cooling-water bath were held continuously to prevent thermal effect during the degradation process and tokeep the uniformity. A 5W LED with emission wavelength of 365 ± 5 nm and a 300 W xenon lamp with 420 nm cut-off filters were used as the UV (365~800 nm) and visible light sources (420~800 nm), respectively.The incident light source was placed above the aqueous solution vertically, and the illumination intensity for UV and visible lights at upper surface of the solution were about 78 mW/cm^2^ and 132 mW/cm^2^. The photocatalytic processes were conducted under constant temperature, using ice water to cool the system. At the end of regular time intervals, 3 mL suspension was collected and centrifuged, and the residual CR concentrationin the supernatant fluid was analyzed by UV-vis spectrophotometer (UV−3100, Hitachi, Tokyo, Japan). 

## 4. Conclusions

AnNa_2_S chemical bath treatment of Bi_2_O_2_CO_3_ did not generate a second phase. It is shown that the introduction of *S* can effectively broaden the optical absorption range, although it does not apparently change the crystal structure of Bi_2_O_2_CO_3_. The electrons at the top of the valence band in Bi_2_O_2_(CO_3_)_1−*x*_S*_x_* can be excited by shorter wavelengths of sunlight, forming photo-generated electron-hole pairs. This may be due to the formation of chemical bonds between the S^2^^−^ and vacancies on the surface of Bi_2_O_2_CO_3_ crystal, which can affect the surface properties.

Bi_2_O_2_(CO_3_)_1−*x*_S*_x_* can improve the catalytic performance of visible and UV regions to a certain extent by the introduction of *S* in Bi_2_O_2_CO_3_ by chemical bath. This is because the introduction of *S* can effectively suppress the carrier recombination and improve the carrier transport performance. However, *S* can be introduced only into the surface of Bi_2_O_2_CO_3_ by chemical bath at room temperature, and the improvement of catalytic performance is limited.

## Figures and Tables

**Table 1:** Surface area and percentage of crystallinity of the Bi_2_O_2_CO_3_ and M1~M20 powders.

	M0	M1	M2	M5	M10	M20
Surface area (m^2^/g)	0.917	0.973	0.980	1.666	1.823	0.966
Percentage of crystallinity (%)	--	74.43 ± 0.96	65.62 ± 0.61	74.14 ± 0.88	77.31 ± 0.75	79.87 ± 1.75

**Table 2:** EDS quantitative results of the M0~M20 powders.

	M0	M1	M2	M5	M10	M20
C K	43.78	43.88	43.90	44.11	44.30	44.41
O K	45.53	45.30	45.30	45.27	45.17	45.10
S K	--	0.04	0.06	0.11	0.24	0.29
Bi M	10.69	10.78	10.74	10.51	10.29	10.20
*x*	0	0.007	0.011	0.021	0.047	0.057
